# Chromosome Driven Spatial Patterning of Proteins in Bacteria

**DOI:** 10.1371/journal.pcbi.1000986

**Published:** 2010-11-11

**Authors:** Saeed Saberi, Eldon Emberly

**Affiliations:** Physics, Simon Fraser University, Burnaby, British Columbia, Canada; University of Houston, United States of America

## Abstract

The spatial patterning of proteins in bacteria plays an important role in many processes, from cell division to chemotaxis. In the asymmetrically dividing bacteria *Caulobacter crescentus*, a scaffolding protein, PopZ, localizes to both poles and aids the differential patterning of proteins between mother and daughter cells during division. Polar patterning of misfolded proteins in *Escherechia coli* has also been shown, and likely plays an important role in cellular ageing. Recent experiments on both of the above systems suggest that the presence of chromosome free regions along with protein multimerization may be a mechanism for driving the polar localization of proteins. We have developed a simple physical model for protein localization using only these two driving mechanisms. Our model reproduces all the observed patterns of PopZ and misfolded protein localization - from diffuse, unipolar, and bipolar patterns and can also account for the observed patterns in a variety of mutants. The model also suggests new experiments to further test the role of the chromosome in driving protein patterning, and whether such a mechanism is responsible for helping to drive the differentiation of the cell poles.

## Author Summary

A key process in biology is the self-assembly of biomolecules into highly organized structures. This spontaneous assembly can give rise to complex spatial patterns that help give spatial order to the cellular environment. In many bacteria, the patterning of proteins to the cell poles allows the bacteria to differentiate one end of the cell from the other. What mechanisms can lead to the spontaneous organization of proteins to the cell poles? Prior work has shown that such patterning can emerge from interactions between proteins and the cell membrane. In this paper we use computational modeling to show that a novel patterning mechanism involving only the presence of the bacterial chromosome and a self-associating protein is sufficient to generate polar patterning in bacteria. This model explains recent experiments on polar patterning in *C. crescentus* and misfolded protein aggregation in *E. coli* and provides predictions about how this mechanism could spontaneously lead to asymmetric patterning of the poles.

## Introduction

A variety of molecular mechanisms have been identified for localizing proteins in bacteria cells. The emergence of spontaneous patterns from instabilities arising from the reactions of diffusing proteins [1]–[3] and protein polymerization dynamics [4], [5] have been shown to play a role in the patterning of the Min system that regulates cell division [6], [7]. The periodic patterning of protein clusters involved in bacterial chemotaxis is due to the growth of protein domains from purely stochastic nucleation [8], [9]. In many bacteria, proteins that form scaffolds at both poles serve as anchoring points for other localizing proteins and the tethering of the chromosome. Models have shown that membrane curvature can act as a mechanism for generating such polar localization [10], [11] and is indeed responsible for the patterning of the scaffolding protein DivIVA [12], [13]. In all of the above mechanisms, the patterns result from protein-protein interactions and from interactions with the cellular membrane. Recent experiments on the polar localized scaffolding protein, PopZ, in *Caulobacter Crescentus* show that the presence of the chromosome may also play a key organizing role in positioning protein scaffolds at the poles independent of interactions with the membrane [14], [15]. Other recent experimental work has shown that aggregating misfolded protein in the bacteria *Escherechia coli* is also preferentially localized to the poles, in particular to the older mother cell's pole [16], thereby preventing the daughter cell from inheriting potentially deleterious misfolded protein.

**Figure 1:**
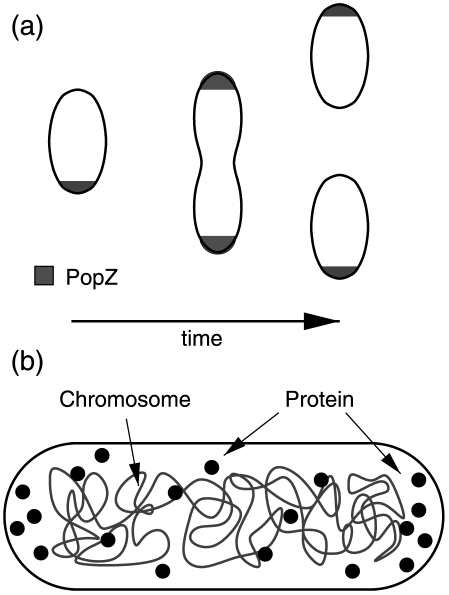
Schematics of observed patterns and model for protein localization in bacteria. (a) Schematic of PopZ localization during cell cycle of *Caulobacter crescentus*. PopZ starts localized in a scaffold at one pole and then during division forms at the 2nd pole. Upon division the two daughter cells both inherit PopZ localized to one pole. (b) Model for protein localization due to nucleoid. Protein freely diffuses throughout the cytoplasm in the presence of the bacterial nucleoid. The nucleoid acts as a region of excluded volume that occupies a space that is smaller than the cellular volume. This creates regions that are empty of DNA at the cell poles. Proteins such as PopZ or misfolded proteins are able to interact with themselves and depending on density can form growing domains in chromosome free regions of the cell.

In *C. crescentus* the scaffolding protein PopZ forms domains that occupy the cytoplasmic space at the two poles [14], [15]. In Fig. 1a a schematic of the dynamic patterning of PopZ over the course of the cell cycle is shown. At the beginning of the cell cycle of *C. crescentus* , PopZ exists at only one pole, inherited from the previous division. After division it begins to assemble and form at the other pole, leading to a bipolar pattern in the dividing cell. However, PopZ can also display a variety of subcellular localizations, not just bipolar, depending on both the amount of PopZ in the cell and on the cellular shape. Amazingly, such patterning can also be reproduced by expressing PopZ in *Escherichia coli* that possesses no *popZ* homologue.

Similar patterning was observed for misfolded proteins in *E. coli*, from unipolar to bipolar localization, with the occasional localization midcell [16]. In these experiments reporter proteins that unfold in response to temperature increases were observed to aggregate at the poles under misfolding conditions. The misfolded protein was consistently found to go to chromosome free regions. When the temperature was lowered and the protein refolded, the domains would fall apart and the protein would return to being diffuse within the cell.

What mechanisms could lead to the diversity of observed patterns of aggregating proteins with bacterial cells, such as PopZ or misfolded protein? Cell curvature has been suggested as a mechanism for sorting proteins to the poles via attraction to lipid domains that prefer negative curvature. Experiments on spherical cells showed that PopZ can show diffuse [14] or localized patterning [15], with the latter arguing against curvature mediated mechanisms. Experiments on non-dividing cells that possess multiple chromosomes showed that PopZ is patterned not only at the poles but also in regions between chromosomes [15]. For the situation of misfolded proteins, it is unlikely that the aggregates are able to sense membrane curvature. As shown, nucleoid occlusion gives a reasonable explanation of the aggregation of misfolded protein in *E. coli*. These experiments have led to the hypothesis that the presence of chromosome free regions along with protein self-association could be a potential mechanism driving localization [15], [16]. This hypothesis requires no active mechanism driving sorting; localization would arise as a result of the entropic forces exerted by the chromosome on the protein and the energy gained from growing protein domains. Prior work on chromosome segregation has shown that purely entropic forces can be sufficient to drive DNA separation within cells without the need to invoke active transport [17]. In [16] a mathematical model of the patterning process using Langevin dynamics for the protein and a mean-field treatment of the nucleoid gave observed patterns. Using a complementary approach we explore a simple biophysical model of the above localization mechanism and whether it may play a role in protein patterning.

In this article we provide a model along with simulations that show that protein multimerization in chromosome free regions can be a sufficient mechanism for polar localization. Fig. 1b summarizes the model. In the cell the chromosome packs into the nucleoid that occupies a significant fraction of the cellular volume, however because of its condensed structure it spends less time exploring the cell poles. Because of this, proteins that multimerize into larger structures, such as PopZ or misfolded protein, will have an entropic force that will naturally sort them to the poles. The protein's ability to nucleate and grow into domains depends upon its density at the poles and also the pressure exerted by the nucleoid on the free volumes at the poles. For fixed nucleoid volume fractions, the protein remains diffuse at low concentrations. At higher concentrations it potentially can nucleate and grow at only one pole and at yet higher concentrations it becomes possible to localize and grow domains at both poles. The model predicts that no other mechanisms besides the formation of chromosome free regions and protein multimerization are required. Using this model we can reproduce all the observed patterns observed for PopZ in *C. crescentus* and misfolded protein in *E. coli* and suggest new experiments that would help to test the model further.

## Results

To model the localization of aggregating proteins in bacterial cells we consider a cellular environment that contains the circular bacterial chromosome and a diffusing protein that is able to self-associate. In bacteria the DNA compacts into a structure called the nucleoid that occupies a smaller volume than that of the cell, and it is estimated to have a volume fraction between 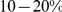 [18], [19]. We model the bacterial DNA as a circular self-avoiding polymer consisting of a tethered string of beads, with a bead size chosen to have a diameter, 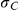, of twice DNA's persistence length 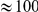 nm. The compacted nucleoid is treated in several different ways: i) as a self-avoiding polymer confined within a smaller subcellular volume as was done in [17], or ii) as a polymer with attractive condensing interactions, but otherwise free to move within the entire cellular volume. The volume fraction of DNA, 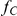, the strength of the interaction between DNA subunits, 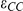 or the size of the subcellular nucleoid volume for the case with no condensing DNA interactions, ultimately control the size and shape of the chromosome. The size and shape of the nucleoid regulates the amount of chromosome free region within the cell that plays an essential role in our model for the patterning of the aggregating protein.

With respect to the aggregating protein, assays on PopZ's structure have revealed that it exists as a multimer and grows as a complex, highly branched three dimensional lattice [14]. Confocal microscopy has revealed that PopZ domains at the poles grow throughout the cytoplasm, and are not just confined to the periphery of the cell membrane [15]. Results on the growth of misfolded protein within bacterial cells reveal similar structures [16]. The volume fraction of cytosolic protein within bacteria has been estimated to be on the order of 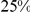 [18]. From the experiments on misfolded protein, patterns emerge when there are 1000s of misfolded protein within the cell that corresponds to a volume fraction 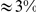 [16]. PopZ is likely to exist in similar amounts. In our model we represent the protein as multimers using beads that are free to move within the cells interior. The diameter of the beads, 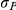, is proportional to the number of proteins making up the multimer, that in principle can differ between different types of aggregating protein, but is chosen to be smaller than the beads making up the DNA polymer. To capture the three-dimensional domain growth of aggregating proteins within bacterial cells we model the interactions between proteins using an attractive potential that allows for isotropic growth in all directions. As will be seen below, there are three key parameters associated with the protein that affect its localization within the cell, its volume fraction 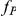 (related to its concentration), the strength of the protein-protein interaction, 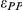 between subunits and the size of the protein multimer beads.

With respect to interactions between the protein and DNA, we assume that there is no interaction other than self-avoidance. Both the DNA polymer and protein are confined within the cell by the cellular membrane that is modeled as a barrier. For further details of the model and the various interactions between the constituents see the Materials and Methods section. We now present results for the patterning of protein in the presence of DNA in different cellular geometries and variation in model parameters.

### Patterns in Cylindrical Cells

Here we consider the patterning of aggregating proteins in cells possessing a cylindrical geometry with length 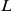 and where the ends are capped with hemispheres of diameter 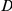. We take the aspect ratio of the cell to be that of an elongating dividing cell, such that 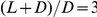. The effects of changing the cellular size and volume fraction of DNA will be presented below. For the bacterial chromosome, we used a volume fraction for DNA of 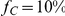, which is consistent with the estimate for *E. coli* from [18] and has been used in other models for bacterial chromosomes [17]. We put attractive interactions between the DNA beads to condense them into a nucleoid (see the figure caption and Materials and Methods for parameter values).

**Figure 2:**
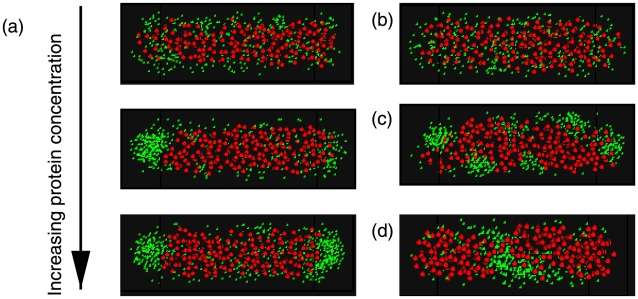
Representative low energy configurations of protein in cylindrical cells. From (a) low concentration of protein (upper) to high (lowest). Protein localization transitions from diffuse (upper, 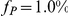) to unipolar (middle, 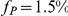), to bipolar (lower, 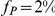) as the concentration increases for fixed DNA volume fraction 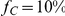. In these figures, the diameter of DNA monomers is 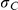, the diameter of PopZ subunits is 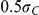, the length of the cell is 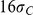 and the diameter is 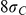. (b) Decreasing the interaction between PopZ monomers, here 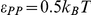, leads to freely diffusing protein monomers. (c) Increasing the protein-protein interaction, 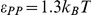, causes protein to form multiple domains. (d) Effect of fragmenting the chromosome into 10 equal fragments. In all the above simulations the nucleoid was modeled using an attractive Lennard-Jones potential with 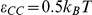.

**Figure 3:**
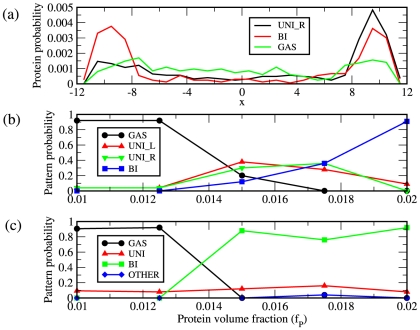
Classifying patterns based on protein distribution in cell. (a) Protein probability density as a function of position along the cell, 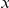 measured in 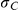. Shown are the densities for diffuse (GAS), unipolar on the right (UNI_R) and bipolar (BI). (b) Shown are the resulting frequencies of protein patterns from 50 separate simulations at each value of 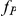 for the cell geometry and DNA density described in Fig. 2a. (c) Same as in (b) except using a DNA volume fraction of 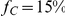. The frequencies of both unipolar patterns have been combined into a single unipolar classification, ‘UNI’ and patterns that result in domains elsewhere than at the poles are classified as ‘OTHER’.

In Fig. 2(a) we show typical protein patterns at different concentrations of aggregating protein. In these simulations, the protein bead diameter is 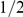 that of the DNA beads. The effect of changing the protein bead size is discussed below. We find that as the volume fraction of protein is changed, different patterns of localization emerge. For low protein concentrations, 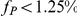, it remains diffuse, mixed throughout the cellular volume (see Fig. 2(a) top panel, and Fig. 3b (black)). The protein's probability density as a function of cell length, 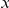, for the diffuse/gas pattern is shown in Fig. 3(a), and it can be seen that it is roughly uniform with position. This is also true for the radial distribution of protein shown in Fig. S1(b), where there is only a slight increase in protein density on the cell's periphery where the chromosomal density is less as shown in Fig. S1(a). As the protein concentration increases, 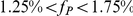, its density becomes sufficient at one pole to seed the formation of a protein domain at one end (Fig. 2(a) middle panel, and Fig. 3a for a representative spatial density of the unipolar phase). Formation of this domain at either pole occurs with equal likelihood (see Fig. 3(b)) since the chance that the chromosome will create a free volume for seed formation is equal for both sides. The overall likelihood is 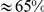 for the unipolar phase at these concentrations, and only rarely does the diffuse state occur. Finally as the protein levels increase further, 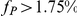, sufficient density builds up to seed the formation of a protein domain at the other pole (see Fig. 2a, bottom panel and Fig. 3b). The localization of protein at the poles is driven partly by the entropy gained by the DNA polymer by forcing protein to the poles and the favorable energy gained from protein multimerization. Formation of domains in locations other than the poles is influenced by factors that we now discuss.

The volume fraction of DNA within the cell affects the types of patterns that can be generated and at what protein concentrations they occur. This effect is particularly relevant since there is a difference between the volume fractions of DNA between *E. coli* and *C. crescentus* due to the fact that their genomes are roughly the same size yet *C. crescentus* has a smaller cellular volume. To separate the effect of DNA volume fraction from cell size, we decided to increase the volume fraction of DNA within the cell from 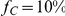 to 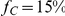 leaving the cell geometry fixed. The results are shown in Fig. 3c for the frequencies of the different patterns as a function of protein concentration. Because of the increased density of chromosome the onset of bipolar patterning is now more abrupt compared to situation with less DNA. Observing the unipolar phase under such conditions becomes less likely as the bipolar phase is favored. Also, there is a small chance of seeing persistent domains at locations other than at the poles (blue curve Fig. 3c). Patterning in wild type *C. crescentus*, favors bipolar patterning of PopZ, whereas the unipolar pattern occurs more frequently in *E. coli*. These results would also predict a potential change from unipolar patterning to bipolar patterning under osmotic shock that would cause the cell size to shrink, thereby increasing the DNA volume fraction [20], [21].

To further test the effect of DNA and its polymer structure in the model, we also consider the possibility of breaking the DNA into smaller fragments. In Fig. 2(d), we show the results for protein localization where the DNA has been broken into 10 fragments and for protein concentrations that previously generated bipolar patterns. Now the protein forms a single extended domain, that from one simulation to the next occurs in different locations. Thus the volume fraction of DNA and it being a connected polymer plays an important role in the localization of the self-associating protein in our model.

We have also modeled the nucleoid using just a self-avoiding DNA polymer confined within a smaller cylindrical volume and find that the similar progression of patterns is found. We find that we need to confine the chromosome to a cylinder whose diameter is 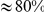 of the cell's diameter, and that for a purely self-avoiding polymer, the volume fraction of DNA that can lead to patterns that emerge over short simulation times is 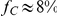. This decrease in DNA volume fraction occurs since the effective excluded volume effect is larger than for the case when there were attractive condensing interactions between DNA beads. Thus it seems that the key mechanism is that there be sufficient chromosome free space to produce a protein density that can seed domain formation, and that different nucleoid models only differ slightly in how they generate this mechanism.

Next we examined the effect of scaling cell size for the situation where unipolar patterns were favored. From the simulations discussed above, using a DNA volume fraction of 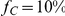 and a protein volume fraction of 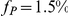 yielded a unipolar in 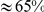 of simulations for cells with a diameter of 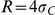. Keeping an aspect of ratio of three we changed the cell radius and computed the probability of observing the various patterns (see Fig. S2a). Only a marginal change in the propensity to form the unipolar pattern was seen, with smaller cells stabilizing the pattern, while in larger cells the diffuse state becomes slightly more likely. Also in the smaller cell geometry it is more likely to see domains formed not just at the poles, and we suspect that these are metastable states. Thus scaling cell size, keeping volume fractions constant for the two components has only a marginal affect on the propensities of patterns. However, reducing the size of the protein diameter from 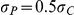 to 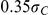 leads to 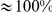 bipolar pattern formation for protein levels that favored unipolar behavior at larger protein size (Fig. S2(c)). Smaller protein multimers have an easier time to find the chromosome free regions at both poles, allowing both poles to form domains equally. Making the protein diameter nearly the same size as the DNA pushes the balance in the opposite direction, favoring the diffuse phase. Thus the size of the intermediate protein aggregates has a significant influence on what patterns are possible.

In our model, the interaction between protein subunits is governed by a single energy, 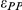, which controls the phase behavior of the protein [22]. In Fig. 2(b,c) we show the effects of either increasing or decreasing 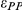. For weak protein interactions, 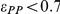 and a concentration that previously led to unipolar localization, we now find that protein returns to being diffuse throughout the cell. This represents the situation of the vast majority of proteins inside a cell that do not possess multimerizing interactions that allow for isotropic domain growth, and so remain diffuse throughout the cellular volume. This was seen in the experiments on misfolded proteins in *E. coli* when the properly folded protein did not localize whereas it did upon unfolding. For strong protein interactions, 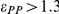, we see the proteins condense into droplets on the periphery of the cell (see Fig. 2c). In Fig. S2b, we show the change in the frequency of various patterns by increasing 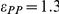. Now the bipolar pattern becomes the most likely, and there are a significant number of times that multi-domain patterns are observed compared to when the interaction was weaker. At these stronger interaction energies, the diffuse pattern is no longer prevalent as the protein always condenses into clusters. Experimental time-lapse images do not show lots of protein domains; rather show rapid turnover of clusters before the final equilibrium pattern stabilizes.

**Figure 4:**
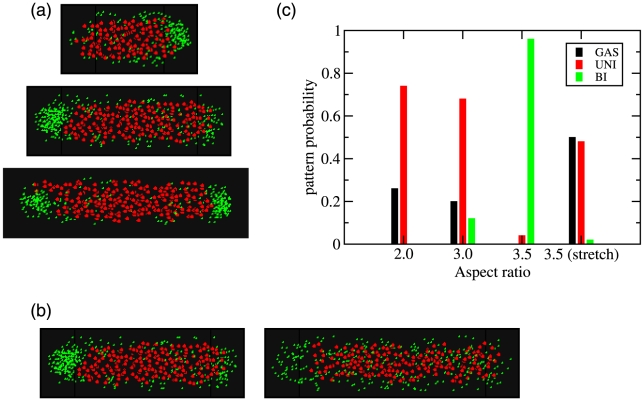
Effect on protein patterning by changing the aspect ratio. (a) In (a), protein and DNA volume fractions are fixed at 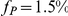 and 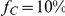 with a cell diameter of 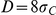. (top) Cell with 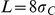 and an aspect ratio of 2.0 and a typical unipolar pattern. (middle) Cell with 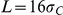 and a aspect ratio of 3.0 showing a unipolar pattern. (bottom) Cell with 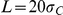, giving an aspect ratio of 3.5 showing the likely bipolar pattern. (b) Affect on patterning by altering cell shape. In (b) the total amount of protein and DNA are fixed using volume fractions are 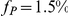 and 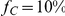 respectively for a cell with 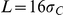 and a diameter of 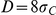. (left) A cell with 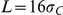 showing unipolar patterning. (right) A cell with 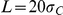 showing a destabilization of the protein domain. For simulations in both (a) and (b) the nucleoid was modeled using an attractive Lennard-Jones potential with 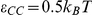 (c) Summary of results for the frequency of the various patterns over 50 simulations at each aspect ratio.

We next consider the situation where the aspect ratio of the cell is changed. First we consider the situation of a growing cell, where through the replication of DNA, the production of protein and by dilution as the cell grows, the volume fractions of both protein and DNA remain fixed. The DNA volume fraction is 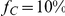 and the protein volume fraction is chosen to be 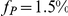 that was found to favor unipolar patterning in a cell with an aspect ratio of three. For smaller aspect ratios, Fig. 4(a-top), the unipolar pattern is favored (see Fig. 4c). As the cell continues to elongate, the unipolar pattern persists, until eventually the bipolar pattern becomes the most likely at larger aspect ratios (Fig. 4(a-bottom)). Lastly we consider changing the aspect ratio by stretching the cell. In Fig. 3(b-left) we show a representative unipolar pattern that emerged for an aspect ratio of three. When the cell is elongated to an aspect ratio of 3.5, with the same initial total DNA and protein amounts, we find that the diffuse state becomes the most likely, (see Fig. 4c and Fig. 4(b-right). Thus we would predict that changing the cellular geometry changes the density of protein at the poles, which is a crucial factor for stable domain growth in our model. We explore other cellular geometries further in the next two sections.

The above simulations were performed with fixed protein concentrations, allowing the system to come to equilibrium from an initially random spatial distribution of protein within the cell interior. We explored the effect of initial conditions by allowing the system to come to equilibrium and then we changed the amount of protein in the cell. In particular this allows us to address whether a cell starting with a protein domain at one pole will continue to grow only at that pole or will a bipolar pattern ultimately emerge as more protein is added? We see the system transition from diffuse to unipolar and then to bipolar localization as the protein concentration is increased from one initial condition to the next. For the diffuse to unipolar transition, unsurprisingly the unipolar pattern emerged with the same frequency as found above using random initial conditions. For the unipolar to bipolar transition, we found that the bipolar pattern was favored although at slightly less frequency than the situation when the initial protein distribution was random (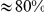 compared to 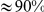 for random initial conditions). Thus under appropriate DNA and protein concentrations it is possible for one polar domain to appear first, and the 2nd pole to form upon addition of more protein. In the case of *C. crescentus* that already has a PopZ domain at one of its poles, in a newly divided cell where the PopZ concentration is likely at levels to satisfy bipolar domain formation, we predict that instead of the preexisting domain continuing to grow, a 2nd polar domain of PopZ will form. The same would also be true for misfolded protein, where if the concentration of misfolded protein is large enough, another aggregate will begin forming at the new pole. Adding more protein to a preexisting bipolar pattern caused the polar domains to grow further, similar to what was seen in PopZ overexpression experiments.

### Patterns in Spherical Cells

In cylindrical cells, where different curvatures of the cell membrane exist, it was speculated that proteins may localize in part due to interactions with biomolecules that sort to the poles because of curvature. Experiments on spherical protoplasts and cells treated with A22 that destabilizes the cytoskeleton leading to spheroid cells showed that these curvature effects may not play a significant part in PopZ localization [15]. In such cells, PopZ was found to be diffuse [14], [15], unipolar [15], and occasionally bi/multipolar [15].

**Figure 5:**
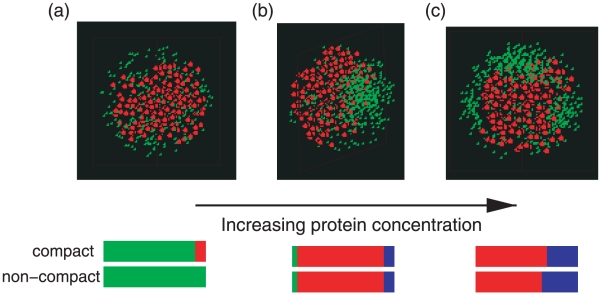
Protein distribution in spherical cells. From left to right, (a) diffuse protein at low concentration (left), 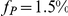 to unispot (center), 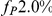 to multi-spot at higher concentrations (right), 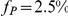. The radius of the cell is 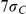, using 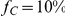 and all other bead sizes and interactions are as given in Fig. 2. Beneath each pattern are shown the frequencies of observing the patterns: diffuse (green), unispot (red), bipolar (blue). Compact refers to a nucleoid modeled using an attractive Lennard-Jones potential with 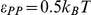 and non-compact is for a nucleoid with only the repulsive portion of the Lennard-Jones potential considered.

Our own simulations involve no specific membrane interactions and yet show patterns of localization of protein, consistent with localization being independent of curvature. In Fig. 5 we show our results on spherical cellular geometries. In these simulations we use the same total cellular volume as was used for the cylindrical cells shown in Fig. 2, the same DNA volume fraction of 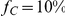. We consider the situation where the nucleoid is condensed using attractive interactions between the DNA beads or decondensed when the attraction between DNA beads is turned off. Interestingly, for protein concentrations that previously had a tendency to form unipolar patterns (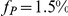) we find only diffuse patterning. At higher protein levels, a single domain is favored, occasionally with multiple smaller protein domains (blue fraction in Fig. 5(b)). For higher protein levels, bipolar patterns become more frequent, opposing each other, and pushing the chromosome into a lobed like structure (see Fig. 5(c)).

For spheroid cells generated using the drug A22 that disrupts the cytoskeleton, it has been suggested that this may serve to destabilize the nucleoid, allowing the chromosome to more fully explore the cell's volume [17]. When we turn off the condensing interaction between DNA beads, leaving just the self-avoidance interaction, and allowing the polymer to explore the full volume of the cell, we do not find a significant affect on the frequencies of the various patterns. There is a slight tendency to favor multiple domains, which has the effect at lower concentrations to keep the system in the diffuse state. But the effects seem marginal.

These results potentially help to explain the observed differences in PopZ localization from two different experiments utilizing A22 to form spheroid cells [14], [15]. For cylindrical *E. coli* cells that favored unipolar spot formation, treatment of A22 leading to spheroid cell geometries showed diffuse behavior [14], consistent with our findings above. We speculate that for the experiment that generated spherical cells [15] that there may have been more time for PopZ to accumulate to levels that admit domain formation. Quantification of protein levels within cells would help to clarify the observed differences to see if it is consistent with our predictions.

### Patterns in Filamentous Cells

**Figure 6:**
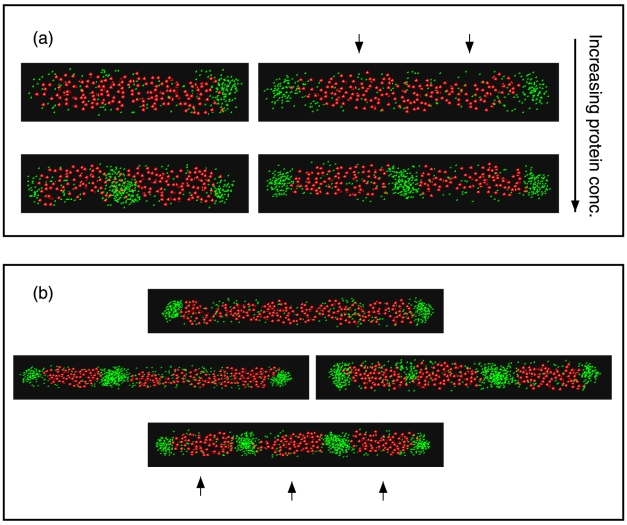
Protein distribution in multi-chromosomal cells. (a) Cell possessing two chromosomes. The cell diameter was taken to be 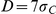 with the length of a single cell having 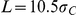, using 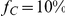 to determine the size of a single chromosome. Protein concentration increases from top to bottom, from 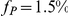 (top) to 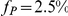 (bottom), and cell's length is varied, from 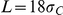 (left) to 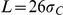 (right). (b) Cell containing three chromosomes using the same individual chromosome size as in (a), with a cell length of 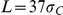. At lower concentrations (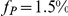), protein forms only at poles (top). At higher protein concentrations, 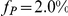 poles and interchromosomal regions can be occupied by protein domains (bottom) and at even higher concentrations (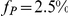) all chromosome free regions can be occupied by a protein domain.

**Figure 7:**
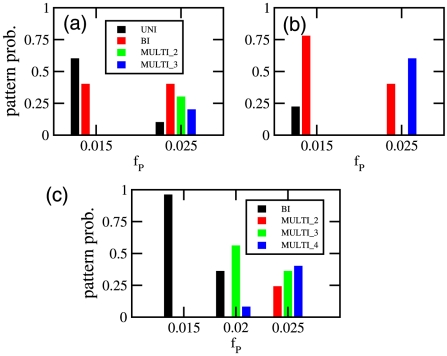
Summary of frequencies of observed patterns in multi-chromosomal cells. Results for a cell possessing two chromosomes with (a) length 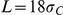 or (b) length 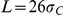. Diameter and DNA volume fraction are as in Fig. 6a. ‘MULTI_2’ corresponds to 2 protein domains, one in between the two chromosomes and one at a pole. ‘MULTI_3’ corresponds to all chromosome free regions being occupied by a protein domain. (c) Results for cells possessing three chromosomes. Here ‘MULTI_2’ are patterns with two protein domains that are not at both poles, ‘MULTI_3’ cells possess three domains and ‘MULTI_4’ cells have all chromosome free regions occupied by a protein domain. Frequecencies of patterns were found as a function of 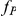 over 25 independent simulations at each value of 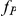 and length, 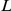.

Experiments on mutants that form filamentous cells possessing multiple chromosomes show that PopZ and misfolded protein not only forms domains at the poles but also at the interchromosomal boundaries [15], [16]. We performed simulations on cells possessing multiple chromosomes and of variable length to see how the protein patterns would change as a function of the length of the cell and the number of chromosomes. The results are shown in Fig. 6 and Fig. 7. In cells possessing two chromosomes and that are less than two full cell lengths; for 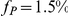 the unipolar pattern is favored (Fig. 7a). For cells that are longer than two full cell lengths, the bipolar pattern becomes favored for the same concentration of protein (see Fig. 6a(top-right) and Fig. 7b). At higher levels of protein, the unipolar pattern is unfavored, and protein domain formation between chromosomes becomes possible (Fig. 6(a), bottom panel). In long filamentous cells, all chromosome free regions can become occupied, and this pattern is favored at higher protein concentrations (see Fig. 6a(bottom-right) and Fig. 7b).

We also simulated cells possessing three chromosomes that now allow for the possibility of two interchromosomal regions (Fig. 6b). In experiments on long cells, not every interchromosomal band was occupied [15]. We find similar behavior, attributing different banding patterns to the concentration of protein (see Fig. 7c). In particular, at certain protein concentrations we find it possible to pattern both poles and one inter-chromosomal boundary (Fig. 6(b-middle)). At yet higher protein concentrations, again all chromosome free regions can become occupied by a protein aggregate. Thus patterns of PopZ in longer cells can be interpreted in the light of a model that only relies on the generation of chromosome free regions and protein multimerization.

## Discussion

Recent work has shown that nucleoid occlusion may be sufficient to drive protein aggregation at the poles [15], [16]. In this paper we have explored a simple biophysical model for how the presence of the nucleoid in addition to multimerizing interactions between proteins such as PopZ or misfolded proteins can localize the protein domains to the poles and interchromosomal regions. Other potential mechanisms, such as membrane curvature may indeed play a role but are not required. As has been pointed out for PopZ [15] and misfolded protein [16] the spontaneous organization of a protein to the poles depending on concentration has a number of biologically attractive outcomes. In particular, the model showed that under appropriate cell geometries and DNA concentration, it is possible for the pattern to transition from diffuse, to unipolar to bipolar with increasing protein concentration. Breaking the spatial symmetry provides the opportunity to differentially pattern the polar regions. Thus there is no requirement for any prior history to differentiate the poles as the breaking of spatial symmetry due to the formation of the unipolar pattern can occur spontaneously.

The modeling presented here may help to interpret some of the recent experimental findings. In particular, recent experimental work has shown that by treating cells with the drug A22 that destabilize the cytoskeleton through action on the cytoskeletal protein MreB, producing spheroidal cells, can lead to either diffuse [14] or localized PopZ [15]. Our model would offer a resolution to these results, suggesting that the observations are consistent with the systems having differing PopZ levels - diffuse at lower concentrations and localized at higher. Further experimental characterization of PopZ levels is required to determine whether a difference in total amounts could account for the difference in observed patterns.

Another connection to experiment is with respect to the cell cycle and the effect of initial conditions on the emergent protein patterns. For a cell with a bipolar pattern, upon division two unipolar cells result, yet protein levels should be at the same concentration. We found that cells that start with a unipolar initial condition, but with concentrations that admit the formation of a bipolar pattern, do indeed have the bipolar pattern emerging as the most frequent. Overexpression experiments of PopZ showed continued growth of both polar domains, and our results are consistent with these findings, in that protein that is added to a bipolar initial condition favors continued growth at both poles.

Experiments on the aggregation of misfolded protein in *E. coli* showed that when the protein was allowed to refold, the domains disappeared and the protein went back to being diffuse within the cell. We found this when we lowered/turned off the attraction between protein monomers. We also found that patterns could be destabilized via mechanical manipulation of the cells. Doing such experiments on the misfolded protein in *E. coli* system seems like a reasonable test. Such experiments on PopZ may be hindered by potential domain stabilizing interactions with membrane bound protein like SpmX that is known to interact with PopZ. Such interactions could help tether PopZ to the membrane thereby stabilizing growing domains.

Our results also may help to provide some insight into the differences in patterning observed between *C. crescentus* and *E. coli*. *E. coli* cells are larger than *C. crescentus* yielding a lower volume fraction of DNA given that their genomes are roughly the same size. In experimental work, unipolar patterns were more often seen in the ectopic expression of PopZ in *E. coli*, and were also observed when protein misfolding was induced. Under wild-type or overexpression conditions of PopZ in *C. crescentus* bipolar patterning was favored. Our own results show that for increased DNA volume fractions, there is an abrupt transition from the diffuse pattern to bipolar pattern as the protein concentration is increased, with the unipolar pattern only rarely occurring. Thus the model would predict that the increased volume fraction in *C. crescentus* favors it forming bipolar patterns whereas similar PopZ levels in *E. coli* would favor unipolar patterns. These results also suggest new experiments using osmotic shock to change cell size thereby changing the volume fraction of DNA. For *E. coli* cells with a unipolar pattern, the model would predict that shrinking cell size, thereby increasing DNA volume fraction would favor bipolar patterns.

Although our model is simple the observed protein fractions that are seen to lead to patterning in experiment (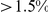 volume fraction for misfolded protein, corresponding to 1000s of proteins) are consistent with the values seen in our model. In our model, patterns emerge when there are several hundred protein beads, where each bead represents an aggregate of 6–15 proteins, thus yielding total protein amounts in the thousands. Experimental data on PopZ suggest that there are likely 1000s of PopZ proteins in the cell. The ratio of bead size between DNA and the protein multimer had a strong influence on the pattern and we expect that scaling of this ratio should lead to similar pattern formation. Using a sphere to represent a segment of DNA likely overestimates its excluded volume, hence using cylindrical segments with a smaller crossectional area would admit using smaller bead sizes for the protein multimers, yet with similar patterns emerging.

Besides changing the volume fraction of DNA, another suggestion for an experiment would be to damage the DNA such that it is fragmented within the cell. We predict that breaking the chromosome into fragments should be sufficient to destroy the polar patterning and that protein localization should then occur at random positions within the cell volume. Deforming the cells is also predicted to have a significant effect on protein patterning; elongating the cell is predicted to destabilize domains. It is also predicted that for a fixed protein and DNA volume fraction that as the aspect ratio grows there should be an abrupt change with bipolar patterns being favoured for aspect ratios 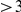. Careful control of the concentration of aggregating protein in the cell and monitoring the resulting patterns as it grows should show provide a test of these predictions.

Despite its simplicity, having only three molecular parameters and the cellular geometry, the model has rich behavior. It has connections to cluster growth models in phase separating systems [23] and extending such theory to include the physics of the confined DNA polymer [24] is forthcoming. The simulations presented here have been at equilibrium, showing the most likely low energy conformations. A dynamical treatment, taking into account diffusion and reaction kinetics as was done in [16] will provide insight into the time-scale of formation of the domains and how this relates to the domain kinetics seen in experiment. This will be addressed in future work.

In summary, recent experiments on the polar localization of aggregating proteins suggest that patterning is driven by protein self-association in regions free of DNA. We have shown that a model based on such a mechanism is indeed sufficient to produce all the variety of observed patterns. Its simplicity is attractive as it requires no active components; the patterns spontaneously emerge via a competition between the entropy of the chromosome and the energetic gain of forming a protein domain.

## Model

The cell is modeled as a closed volume of either i) cylindrical geometry with a cylindrical region of length 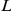 capped by hemispheres of diameter 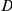, or ii) a sphere with radius 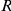. Inside the cellular volume there is the chromosome and diffusing beads representing protein multimers. The chromosome is modeled as a circular string of tethered self-avoiding beads. The diameter of each bead is given by 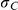 and the number of beads making up the chromosome, 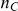 is calculated from its volume fraction, 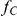 such that 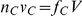 where 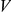 is the volume of the cell, and 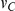 the volume of a DNA bead. All length scales in the system are expressed in terms of the bead size of DNA, which is taken to be 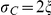 where 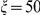 nm is the persistence length of DNA. For a cell geometry of 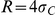 and 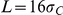, and a DNA volume fraction of 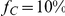, this gives a chromosome consisting of 204 beads. The largest chromosome modeled was for a geometry of 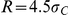 and 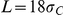 giving 292 beads making up the chromosome. Protein multimers are modeled as beads with diameter 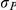, and their number is given by 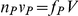, where 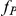 is the volume fraction of protein. For 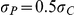 and a cell with 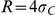 and an aspect ratio of 3.0, the amount of protein multimers in the cell ranges from 164 for 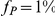 to 328 for 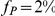. For the larger cell geometries there are 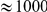 protein multimers in the cell.

With respect to energetic interactions, for the beads making up the chromosome, they are tethered together using the following potential between neighboring beads, 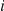 and 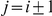,
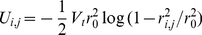
(1)where 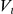 is the tethering strength and 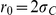 sets the length scale of the potential. We take 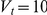 that keeps the beads on the DNA chain from stretching much beyond a bead-to-bead distance of 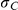.

We use a Lennard-Jones potential to model the interactions between the various types of beads in the system, given by
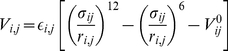
(2)where 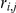 is the distance between beads 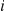 and 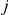, 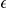 is the interaction strength and has three possible values depending on the interaction type, 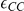, 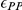 and 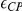 for DNA-DNA, protein-protein and DNA-protein respectively, 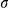 is the interaction distance and again has three possible values, 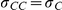, 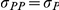 and 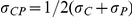 and 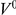 is either 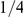 or 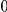 depending on whether the potential function is cut-off and purely repulsive, or attractive. For the former, the interaction between beads is only evaluated if 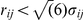. The interaction between bead 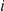 and the cell wall is modeled using a cut-off Lennard-Jones potential, where the 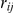 in the above equation is replaced by 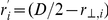 where 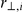 is the perpendicular distance between the bead and the wall. Unless specified elsewhere in the text all 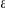 were set to be 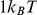 except for 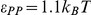.

The system of DNA and aggregating protein is simulated using Metropolis Monte Carlo (MC). Each simulation starts with the circular DNA in a stretched out configuration in a long cell and is allowed to equilibrate as the cell contracts to its final volume. After the chromosome has equilibrated within the cell's final volume, protein beads are added to the cell randomly. Both protein and the DNA have a fixed amplitude step size set to 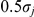, with 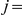
P, or C respectively.

We use a heuristic procedure to define protein patterns. At the end of each simulation, the final configuration of beads is used to calculate the density of protein along the length of the cell. To define the pattern, the cell was divided into equal sized bins of width equal to the diameter of the cell. If the protein density anywhere within a bin exceeded a predefined cutoff (we used a value of 0.002), then a protein domain was deemed to exist in that region. Patterns were then classified as either gas, unipolar-left/right/middle, bipolar or multidomain if more than two domains exist.

## Supporting Information

Figure S1 Radial probability densities for (a) DNA and (b) protein. Cell geometry was as in Fig. 2a, with a cell radius of R = 4σ_C_ using a protein volume fraction of f_P_ = 1.0% that gives diffuse behaviour, leading to well mixed protein throughout the cellular volume. In (a) the density of DNA beads as a function of radius is shown for a nucleoid model that has attractive Lennard-Jones interactions (black) or is a cut-off, purely self-avoiding Lennard-Jones potential (red). In (b), the radial protein density is shown for the above two nucleoid models and for the case of smaller protein diameter size, in this case σ_P_ = 0.35 σ_C_. In all cases, there is a significant protein density in the nucleoid interior with a slight increase nearer to the membrane.
(0.02 MB EPS)

Figure S2 The frequencies of protein spatial patterns as a function of the model's parameters. In this case ‘OTHER’ represents patterns that do not fall into either unipolar or bipolar cases. In all simulations the volume fraction of DNA is f_C_ = 10% and protein is f_P_ = 1.5% (a) Changing cell radius, keeping an aspect ratio of three. (b) Increasing the protein-protein interaction strength from ε_PP_ = 1.1 to ε_PP_ = 1.3. (c) Affect of making the protein diameter smaller from σ_P_ = 0.5 σ_C_ to 0.35 σ_C_. (d) Affect of making protein diameter larger from σ_P_ = 0.5 σ_C_ to 0.7 σ_C_.
(0.02 MB EPS)

## References

[1] Loose M, Fischer-Friedrich E, Ries J, Kruse K, Schwille P (2008). Spatial regulators for bacterial cell division self-organize into surface waves in vitro.. Science.

[2] Howard M, Rutenberg A, de Vet S (2001). Dynamic compartmentalization of bacteria: accurate division in e. coli.. Phys Rev Lett.

[3] Huang K, Meir Y, Wingreen N (2003). Dynamic structures in escherichia coli: spontaneous formation of mine rings and mind polar zones.. Proc Natl Acad Sci U S A.

[4] Szeto T, Rowland S, Rothfield L, King G (2002). Membrane localization of mind is mediated by a c-terminal motif that is conserved across eubacteria, archaea, and chloroplasts.. Proc Natl Acad Sci U S A.

[5] Cytrynbaum E, Marshall B (2007). A multistranded polymer model explains minde dynamics in e. coli cell division.. Biophys J.

[6] Raskin D, de Boer P (1999). Rapid pole-to-pole oscillation of a protein required for directing division to the middle of escherichia coli.. Proc Natl Acad Sci U S A.

[7] Hu Z, Lutkenhaus J (1999). Topological regulation of cell division in escherichia coli involves rapid pole to pole oscillation of the division inhibitor minc under the control of mind and mine.. Mol Microbiol.

[8] Greenfield D, McEvoy A, Shroff H, Crooks G, Wingreen N (2009). Self-organization of the escherichia coli chemotaxis network imaged with super-resolution light microscopy.. PLoS Biol.

[9] Wang H, Wingreen N, Mukhopadhyay R (2008). Self-organized periodicity of protein clusters in growing bacteria.. Phys Rev Lett.

[10] Huang K, Mukhopadhyay R, Wingreen N (2006). A curvature-mediated mechanism for localization of lipids to bacterial poles.. PLoS Comput Biol.

[11] Howard M (2004). A mechanism for polar protein localization in bacteria.. J Mol Biol.

[12] Ramamurthi K, Losick R (2009). Negative membrane curvature as a cue for subcellular localization of a bacterial protein.. Proc Natl Acad Sci U S A.

[13] Lenarcic R, Halbedel S, Visser L, Shaw M, Wu L (2009). Localisation of diviva by targeting to negatively curved membranes.. EMBO J.

[14] Bowman G, Comolli L, Zhu J, Eckart M, Koenig M (2008). A polymeric protein anchors the chromosomal origin/parb complex at a bacterial cell pole.. Cell.

[15] Ebersbach G, Briegel A, Jensen G, Jacobs-Wagner C (2008). A self-associating protein critical for chromosome attachment, division, and polar organization in caulobacter.. Cell.

[16] Winkler J, Seybert A, Konig L, Pruggnaller S, Haselmann U (2010). Quantitative and spatio-temporal features of protein aggregation in escherichia coli and consequences on protein quality control and cellular ageing.. EMBO J.

[17] Jun S, Mulder B (2006). Entropy-driven spatial organization of highly confined polymers: lessons for the bacterial chromosome.. Proc Natl Acad Sci U S A.

[18] Woldringh C, Odijk T, Charlebois R (1999). Structure of dna within the bacterial cell: physics and physiology..

[19] Odijk T (1998). Osmotic compaction of supercoiled dna into a bacterial nucleoid.. Biophys Chem.

[20] Woldringh C, Binnerts J, Mans A (1981). Variation in escherichia coli buoyant density measured in percoll gradients.. J Bacteriology.

[21] Koch A (1984). Shrinkage of growing escherichia coli cells by osmotic challenge.. J Bacteriology.

[22] Hansen J, Verlet L (1969). Phase transitions of the lennard-jones system.. Phys Rev.

[23] Sagui C, Grant M (1999). Theory of nucleation and growth during phase separation.. Phys Rev E.

[24] Jun S, Arnold A, Ha BY (2007). Confined space and effective interactions of multiple self-avoiding chains.. Phys Rev Lett.

